# Intraspecific genetic variation in *Trichinella spiralis* and *Trichinella britovi* populations circulating in different geographical regions of Poland

**DOI:** 10.1016/j.ijppaw.2019.07.009

**Published:** 2019-07-31

**Authors:** Ewa Bilska-Zając, Frits Franssen, Mirosław Różycki, Arno Swart, Jacek Karamon, Jacek Sroka, Jolanta Zdybel, Anna Ziętek – Barszcz, Tomasz Cencek

**Affiliations:** a Department of Parasitology and Invasive Diseases, National Veterinary Research Institute in Pulawy, Al. Partyzantow 57, 24-100, Pulawy, Poland; b National Institute for Public Health and the Environment (RIVM), Bilthoven, the Netherlands; c Department of Epidemiology and Risk Assessment, National Veterinary Research InstitutePulawy, Al. Partyzantow 57, 24-100, Pulawy, Poland

**Keywords:** T. spiralis, T. britovi, Genetic variability, Spatial analysis, PCA analysis

## Abstract

*Trichinella spiralis* and *Trichinella britovi* are species of nematodes which are responsible for the majority of *Trichinella* infections in the world and the most prevalent in Poland. The most abundant species – *T. spiralis,* is considered to be more genetically homogeneous in Europe than *T. britovi*. The aim of the present study was to determine the genetic variability in *T. spiralis* and *T. britovi* populations based on nuclear 5S rDNA intergenic spacer region (5S rDNA) and cytochrome c oxidase 1 (COX1) gene sequences. For the study, 55 isolates of *T. spiralis* and 50 isolates of *T. britovi* isolated from wild boars, pigs, brown rat and a red fox were analyzed. Based on the analysis of both genes, the genetic variability within populations of *T. spiralis* and *T. britovi* differed. In *T. spiralis*, two single nucleotide polymorphisms (SNPs) were observed in the 612 bp 5S rDNA gene fragment, and one SNP was detected in the 700 bp COX1 gene fragment. In *T. britovi*, 17 single nucleotide variations (SNVs) were detected in the 5S rDNA gene fragment (among them 16 SNPs), while COX1 sequence analysis revealed the occurrence of 20 SNVs between the sequences tested (among them 19 SNPs).

For the majority of *T. spiralis* isolates the investigated larvae presented uniform haplotypes. In contrast, most of the isolates of *T. britovi* consisted of larvae of different haplotypes. Geographical analysis showed that each region exhibited different haplotype composition and richness. Warmińsko-Mazurskie and Zachodniopomorskie regions were the richest in haplotypes (15 and 16 haplotypes, respectively). We used heatmaps showing a characteristic pattern for each region graphically. This may allow to differentiate regions based on the occurrence of particular haplotypes. Furthermore, a PCA analysis on the SNP level yielded biplots that show that certain haplotypes/genotypes are associated with (clusters of) regions.

## Highlights

• The genetic variability within the Polish *T. britovi* population is high*.*
• 5S rDNA and COX1 variability in *T. spiralis* is low compared to *T. britovi.*
• *T. britovi* variability may correlate to geographical origin.
• Heatmap and PCA analysis could be helpful analytical tool for epidemiological investigations.

## Graphical abstract

**Figure:**
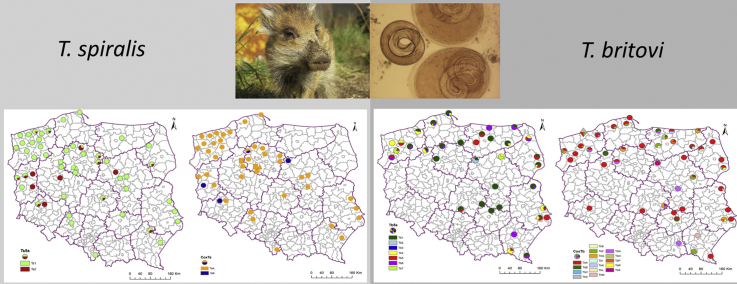

## 1 Introduction

The majority of *Trichinella* infections of animals in Poland are caused by *Trichinella spiralis* and *Trichinella britovi.* Both species occur at different prevalence, depending on the host. More than 75% of infections in wild boars are caused by *T. spiralis*, while in red foxes mainly *T. britovi* is found (Bilska-Zajac et al., 2013; Cabaj et al., 2000). In humans, both species can cause trichinellosis. The species identification of *Trichinella* muscle larvae is most commonly performed using multiplex PCR, a method that easily allows species recognition (Zarlenga et al., 1999). However, multiplex PCR is not suitable to detect intraspecific genetic differences in *Trichinella* species. Molecular analysis using the 5S rDNA intergenic spacer region (5S rDNA) and cytochrome c oxidase 1 (COX1) appears more accurate for this purpose, which previously evidenced the occurrence of genetic variation in both *T. spiralis* and *T. britovi* populations (Franssen et al., 2015). Studies on genetic differences within *Trichinella* species is scant and concerns various genes, and few studies have indicated the existence of intraspecific genetic variability (Odoyevskaya & Spiridonov, 2015; Webb and Rosenthal, 2010; Rosenthal et al., 2008). Identifying the level of genetic variation within *Trichinella* species could aid in finding specific mutation characteristic for given geographical regions. This could be used for source attribution following outbreaks of trichinellosis.

The aim of the present study was to evaluate the genetic variability in *T. spiralis* and *T. britovi* populations occurring in Poland in wild boar, which is the main source of trichinellosis in humans, based on nuclear 5S rDNA and mitochondrial COX1. Additional isolates obtained from other hosts (domestic pigs, red fox and brown rat) were included in the present study.

## 2 Material and methods

In the present study, 105 isolates of *Trichinella* spp. were used. The nematodes in each isolate were first identified to species level by multiplex PCR using 5 individual larvae per isolate. In total, 55 isolates of *T. spiralis* and 50 isolates of *T. britovi* were collected, mainly from wild boars (*Sus scrofa*) (n = 99), domestic pigs (*Sus scrofa f. domestica*) (n = 4), one brown rat (*Rattus norvegicus*) and one red fox (*Vulpes vulpes*) (Table 1).

**Table 1:** Number and origin of isolates of *T. spiralis* and *T. britovi* used in the study (wb – wild boar, p – pigs, f – red fox, r – rat).

Region	No of isolates	
	*T. spiralis*	*T. britovi*
Dolnośląskie	2 wb	1 wb
Kujawsko – Pomorskie	7 wb, 3p, 1 r	2 wb
Lubelskie	3 wb	5 wb
Lubuskie	5 wb	1 wb
Łódzkie	1 wb	2 wb
Małopolskie	-	1 wb
Mazowieckie	5 wb	5 wb
Opolskie	2 wb	-
Podkarpackie	2 wb	4 wb
Podlaskie	2 wb	5 wb
Pomorskie	2 wb	5 wb
Śląskie	1 wb	1 wb
Świętokrzyskie	1 wb	2 wb
Warmińsko – Mazurskie	1 wb	7 wb
Wielkopolskie	3 wb, 1 p	2 wb
Zachodniopomorskie	13 wb	6 wb, 1 f
Total	55	50

### 2.1 DNA extraction

From each larva DNA was extracted, using DNA IQ System Kit (Promega, USA) according to the manufacturer's protocol. Isolated DNA was stored frozen at −20 °C until further use.

### 2.2 PCR reactions

Amplification of the 5S rDNA gene fragment was performed in a PCR mix consisting of 5 μl of 10X Taq Buffer supplemented with 500 mM KCl (ThermoScientific, USA), 6 μl (25 mM) MgCl_2_, 5 μl deoxynucleotide mix (dATP, dCTP, dGTP, dTTP at 0.2 mM concentration), 1 U of thermostable Taq polymerase (ThermoScientific, USA), oligonucleotide primers according to Rombout et al. (2001) (Table 2), nuclease free water added up to a final volume of 50 μl, to which 5 μl of template DNA was added.

**Table 2:** Primers for amplification of 5S rDNA and COX1 gene.

Primer	Sequence (5’→3′)	Amplified gene	Amplicon size [bp]	Primer concentration [μM]
5SFOR	GCGAATTCTTGGATCGGAGACGGCCTG	*5S rDNA*	~750	1
5SRev	GCTCTAGACGAGATGTCGTGCTTTCAACG			
Cox1For	TACCTATACTACTAAGAGGATTCGGA	*COX1*	~760	1
Cox1Rev	CTAGTACTCATAGTATGGCTGGTG

**Table 3:** Thermocycler parameters for 5S rDNA and COX1 gene amplification.

	Predenaturation	40 cycles			Elongation
		Denaturation	Annealing	Elongation	
	Temp./Time [min]				
5S rDNA PCR	95 °C/15	95 °C/0,5	48 °C/1	72 °C/1,5	72 °C/10
COX1 PCR	95 °C/15	95 °C/1	50 °C/1	72 °C/1,5	72 °C/10

Amplification of the COX1 gene was performed in a PCR mix consisting of 30 μl of GoTaq G2 Master Mix (Promega, USA), 0.5 μl of 10 mM oligonucleotide primers according to Franssen et al. (2015) (Table 2), nuclease-free water added up to a final volume of 50 μl, to which 4 μl of template DNA was added. For each PCR reaction, a negative control (nuclease free water), positive control (template DNA from reference strains of *Trichinella* ISS336, ISS324, form EURLP, https://trichinella.iss.it/) and reaction control (template DNA from *Anisakis simplex*) was included. The PCR reactions (5s rDNA and COX1) were carried out in thermocycler TProfessional (Biometra) under conditions according to Table 3.

5S rDNA and COX1 PCR amplicons from all selected samples were separated by horizontal electrophoresis in 2% agarose gels stained by Simply Safe (Eurx, Poland). Then, the amplification products were selected for sequencing, based on molecular size. Sequencing was performed by Sanger sequencing at a commercial company (Genomed S.A., Warsaw, Poland).The resulting nucleotide reverse and forward sequences (chromatograms) of 5S rDNA and COX1 gene fragments were edited manually and analyzed in Geneious R7 (Kearse et al., 2012). Obtained consensus sequences were aligned with reference sequences (GenBank accession numbers AY009946, GU325737, KP900345, KP900334, KM357413.1, NC025750, KM357420.1, KJ716693 and KP900334) and screened for the presence of single nucleotide variations (SNV) and/or single nucleotide polymorphism (SNPs) using program settings in Geneious R7 (Kearse et al., 2012). Next, based on determined SNVs and SNPs, the genotypes based on 5S rDNA partial gene sequence and haplotypes based on COX1 partial gene sequence were determined.

### 2.3 Phylogenetic analysis

The phylogenetic analysis of the obtained variants was performed in MEGA X. The evolutionary history was inferred using the Maximum Parsimony method. The MP tree was obtained using the Subtree-Pruning-Regrafting (SPR) algorithm with search level 0 in which initial trees were obtained by random addition of sequences (100 replicates). Bootstrap values were calculated from 10,000 replicates (Nei & Kumar, 2000; Kumar et al., 2018). The trimmed COX1 sequences were translated into amino acid sequences, aligned and analyzed in Geneious R7 software (Kearse et al., 2012).

### 2.4 Statistical and geographical distribution analysis

Genetic variation in combination with geographical data were analyzed using ‘heatmap3’ and PCA generalized linear modelling in R version 3.5.1 (2018-07-02) (R Core Team, 2014). Geographical distribution analysis was performed using ArcGIS 10.4.1 (ESRI).Collected data were mapped with ArcGIS 10.4.1 (ESRI).

Instead of studying the distribution of geno/haplotypes over regions, an alternative is to consider differences on the level of the single nucleotide polymorphism (SNP). To this end we created a table with region in rows, and SNP positions (for both 5S and COX1) in columns; entries consist of total number of SNPs. Subsequently, each row was normalized to 100%, which allowed entries to correspond to the proportion of a certain SNP in a region. This amounts to a representation which is not yet very insightful and, hence, we processed the data using a principal component analysis (PCA) to transformed axes (PC1 and PC2), which show the maximum amount of variation in the data.

## 3 Results

### 3.1 Sequencing results

#### 3.1.1 5S rDNA

PCR of the 5S rDNA gene fragment resulted in amplification products of 750 bp in length which were obtained from 258 *T. spiralis* larvae (from 55 isolates) and 240 *T. britovi* larvae (from 50 isolates). No amplification products were obtained for DNA samples isolated from 27 larvae. These individuals were excluded from further analysis. From all obtained amplicons, 200 *T. spiralis* consensus 5S rDNA sequences remained to be used for analysis, from a total of 55 isolates; 198 *T. britovi* consensus 5S rDNA sequences remained to be used for further analysis from a total of 44 *T. britovi* isolates. The 612 bp alignment of all 5S rDNA consensus sequences of *T. spiralis* with reference sequence from the GenBank database (AY009946) showed two single nucleotide polymorphisms (SNPs). Both mutations were transition of adenine to guanine. The first mutation was situated at position 433 and the second at 436 (Table 4).

**Table 4:**
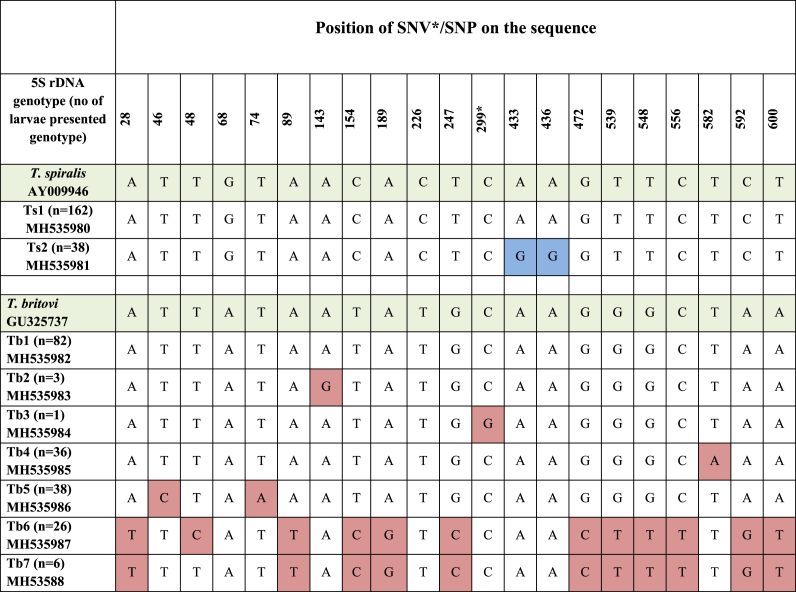
Single nucleotide variation/polymorphism detected 5S rDNA partial sequences of *T. spiralis* and *T. britovi* (* - SNV).

The comparison revealed two genotypes of *T. spiralis*, designated genotype 1 (Ts1), identical to the reference sequences from GenBank and genotype 2 (Ts2), characterized by the occurrence of two simultaneous point mutations. The Ts1 genotype was more frequent. Nucleotide sequences of each genotype were submitted to GenBank (Table 4).

**Fig. 1:**
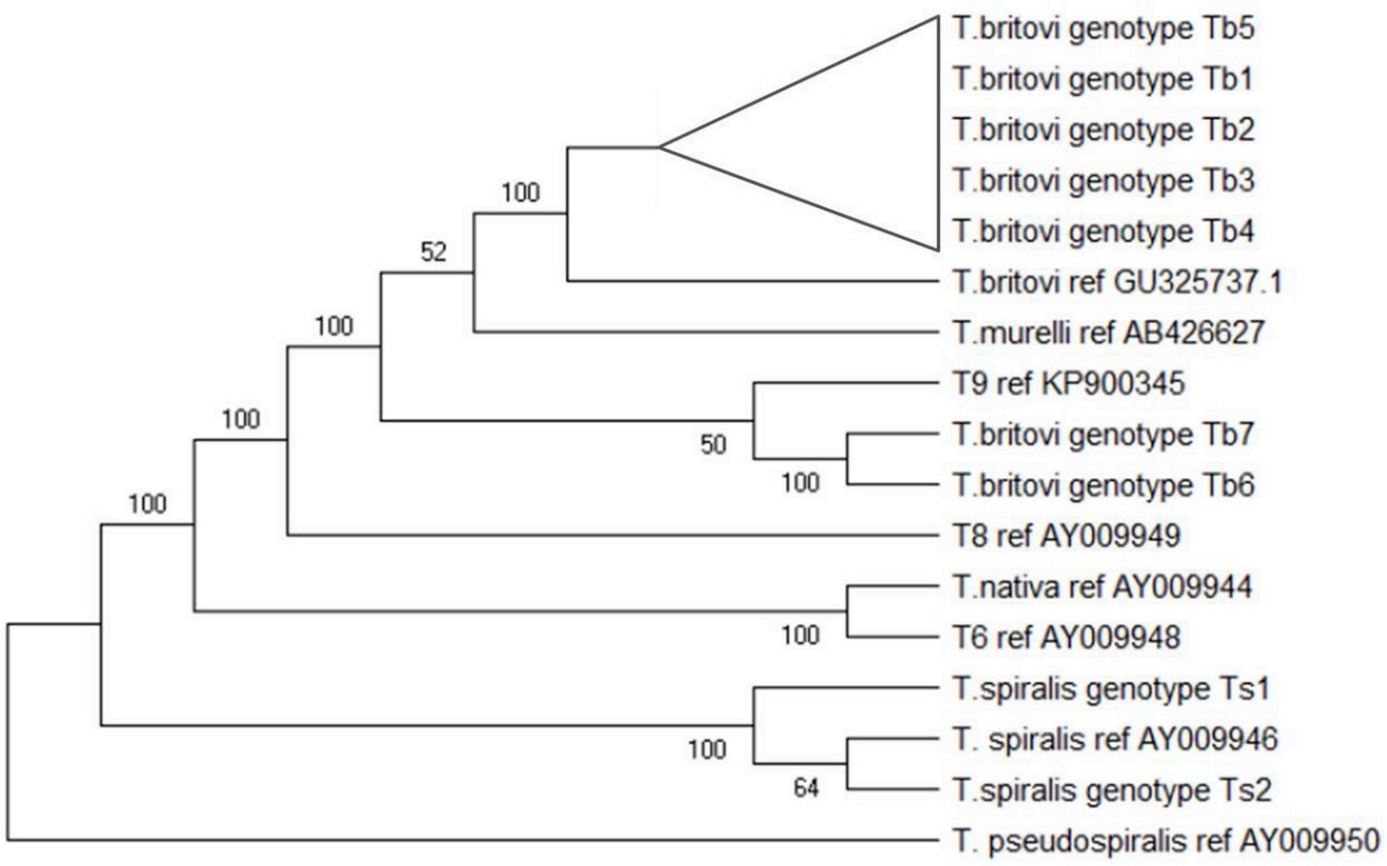
**Maximum Parsimony analysis based on 5S rDNA nucleotide sequences.** The tree shows a clear separation of *T. spiralis* from its descendants. Reference sequences are indicated by their respective Genbank accession codes. *T. britovi* genotypes Tb6 and Tb7 cluster with *Trichinella* genotype T9, whereas the other *T. britovi* haplotypes cluster with *T.murelli*. The non-encapsulated species *T. pseudospiralis* served as outgroup. Bootstrap values were derived from 10,000 replications.

The alignment of 5S rDNA consensus sequences of *T. britovi* with a *T. britovi* reference sequence from GenBank (GU325737) revealed 17 single nucleotide variations (SNVs), of which 16 were identify as SNPs (Geneious R7) (Table 4). Mutations were transitions of adenine to guanine, guanine to thymine, thymine to cytosine, cytosine to thymine and transversions of adenine to thymine, thymine to adenine, cytosine to guanine, and guanine to cytosine. Based on the occurrence of discovered single nucleotide differences in the analyzed 5S rDNA gene, 7 genotypes were found inside the *T. britovi* population: Tb1, Tb2, Tb3, Tb4, Tb5, Tb6, and Tb7. The highest number of larvae presented genotypes Tb1, Tb5 and Tb4 (Table 4). Nucleotide sequences of following genotypes were submitted to GenBank (Table 4). A Maximum Parsimony tree was inferred from sequences of all obtained genotypes of *T. spiralis* and *T. britovi* along with reference sequences from Genbank as shown in Fig. 1. According to the phylogenetic analysis in the present study, the detected *T. spiralis* genotypes belong to the same clade as the reference sequence *T. spiralis* AY009946. Genotypes Tb1, Tb2, Tb3, Tb4 and Tb5 clustered into one clade, identical to *T. britovi* GU325737. In contrast, genotypes Tb6 and Tb7 clustered into a separate group that was phylogenetically closer to *Trichinella* T9 (KP900345).

#### 3.1.2 COX1 results

The COX1 PCR yielded a product size of 730 bp for both species. The electrophoretic separation of the amplification products showed the presence of a band for 477 samples. The amplicons were obtained from 48 isolates of *T. spiralis* (total of 255 larvae) and 44 isolates of *T. britovi* (total of 222 larvae). No amplification products were obtained from 48 larvae and these samples were excluded from further analysis.

From all obtained amplicons of COX1, 200 consensus sequences from a total of 48 isolates of *T. spiralis* larvae and 192 consensus sequences of *T. britovi* from 44 isolates were available for further analysis.

Alignment of these sequences with the reference sequence of *T. spiralis* from GenBank (KJ716693) resulted in an alignment of 700 bp in length and revealed the presence of one SNP between sequences. The detected mutation was a transition of adenine to guanine in position 216 (Table 5).

**Table 5:**
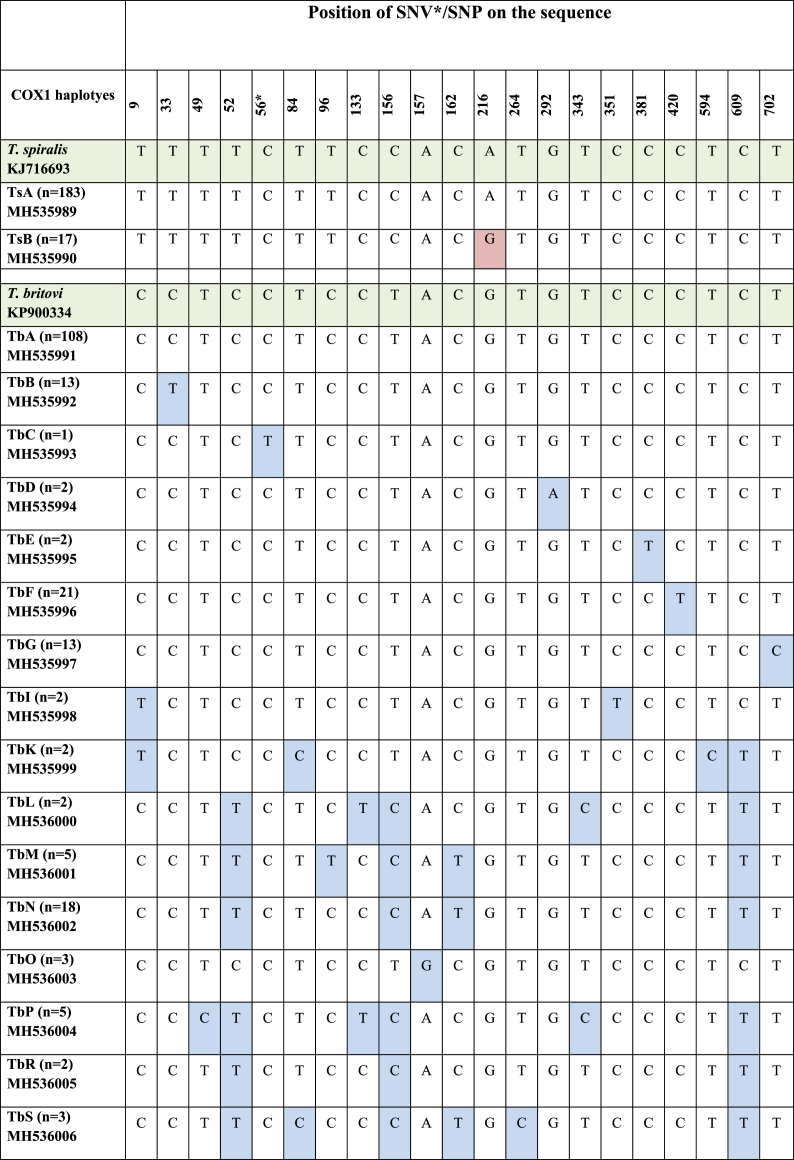
Single nucleotide variation/polymorphism in the alignment of the *T. spiralis* and *T. britovi* COX1 partial gene (* – SNV).

This allowed identification of *T. spiralis* haplotype A (TsA) - identical to GenBank reference sequence KJ716693 and haplotype B (TsB) - characterized by the occurrence of the above mentioned SNP (Table 5). The sequences of both haplotypes were submitted to GenBank (Table 5).

Comparison of COX1 consensus sequences from *T. britovi* isolates with the reference sequence of *T. britovi* (GenBank accesion KP900334), revealed the presence of 20 single nucleotide differences, of which 19 were SNPs in the 710 bp alignment. The SNPs were transitions from cytosine to thymine and vice versa, and adenine to guanine and vice versa. Based on the detected variation, 16 haplotypes of *T. britovi* (TbA, TbB, TbC, TbD, TbE, TbF, TbG, TbI, TbK, TbL, TbM, TbN, TbO, TbP, TbR, TbS) were determined (Table 5). Haplotype TbA was identical to the reference sequence; in the other sequences, one to several SNPs were detected. Haplotypes TbA, TbF and TbN were most abundant haplotypes amongst isolated larvae (Table 5). Nucleotide sequences of haplotypes were deposited to Genbank (Table 5).

**Fig. 2:**
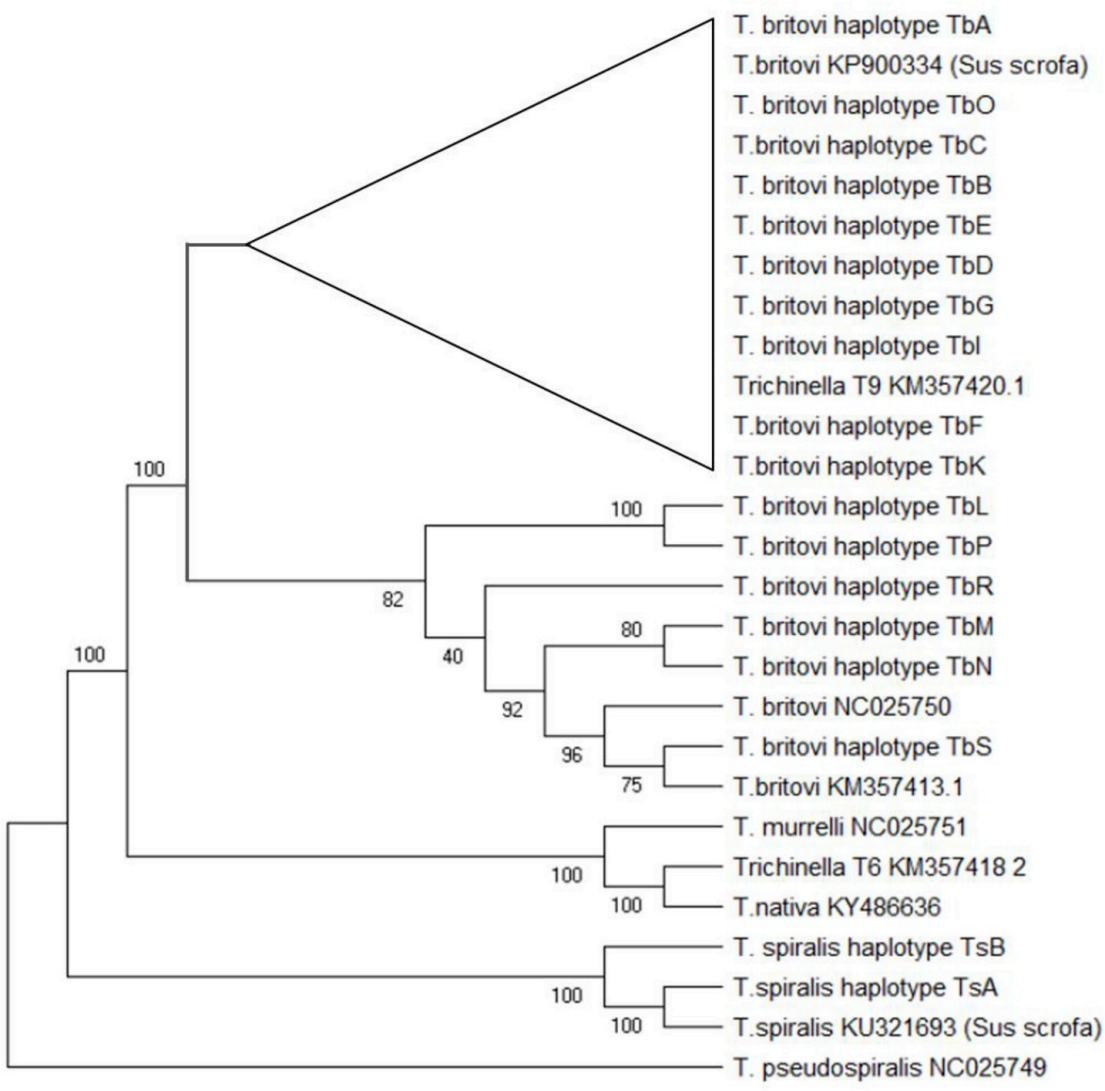
**Maximum Parsimony analysis of taxa based on COX1 nucleotide sequences.** The tree shows a clear separation of *T. spiralis* from its descendants. Reference species sequences are indicated by their respective genbank accession codes. *T. murrelli*, *T. nativa* and *Trichinella* genotype T6 cluster separate from *T. britovi*. There is a clear separation between two *T. britovi* clusters, one of which contains *Trichinella* genotype T9 from Japan. The non-encapsulated species *T. pseudospiralis* served as outgroup. Bootstrap values were derived from 10,000 replications.

A Maximum Parsimony tree was inferred from all obtained haplotype variants of the *T. spiralis* partial COX1 sequences (TsA, TsB), *T. britovi* partial COX1 sequences (TbA, TbB, TbC, TbD, TbE, TbF, TbG, TbI, TbK, TbL, TbM, TbN, TbO, TbP, TbR, TbS) and reference sequences from Genbank (Fig. 2).

*T. spiralis* haplotypes TsA and TsB clustered with Genbank reference KU321693. *T. britovi* haplotypes separated into two clusters, one cluster included haplotypes TbL, -M, –N, -P and –R, and Genbank references KM357413.1 and NC025750. The second cluster contained *T. britovi* haplotypes TbA – G and TbI, -K and –O, and Genbank references KM357420.1 (*Trichinella* T9) and KP900334 (*T. britovi*) (Fig. 2).

Translation of the sequences of COX1 to open reading frames resulted in protein sequences of 240 amino acids in length. The SNP of *T. spiralis* TsB (transition of adenine to guanine in position 216) resulted in a threonine to alanine amino acid replacement in position 72 of the COX1 protein alignment (Table 6). Most of the discovered SNPs in *T. britovi* were silent mutations. Only in three haplotypes, single amino acid substitutions were found respectively at position 98 (isoleucine instead of valine, TbD), position 19 (leucine instead of proline, TbE) and at position 53 (aspartic acid instead of asparagine, TbK) (Table 6).

**Table 6:**
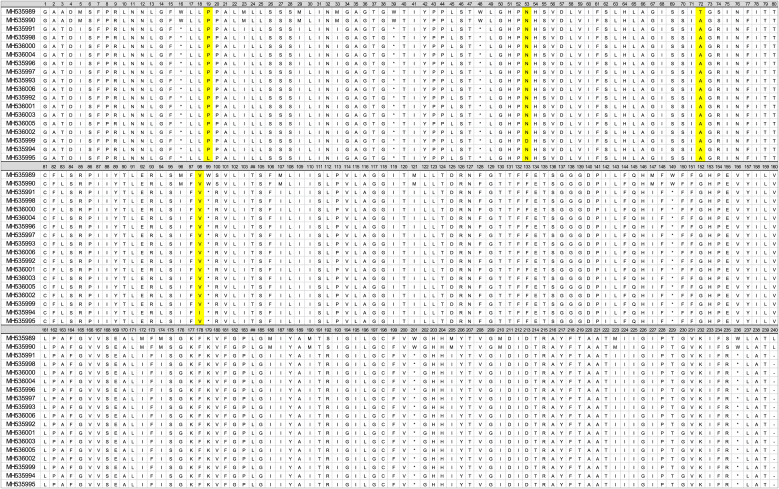
Alignment of the protein sequences of retrieved *T.spiralis* and *T.britovi* haplotypes.

#### 3.1.3 Inter-species hybrids

In the present study, three larvae from three wild boar isolates were recognized as interspecies hybrids of *T. spiralis* and *T. britovi*. Two larvae recognized as *T. spiralis* based on 5S rDNA and multiplex PCR, were identified as *T. britovi* based on their COX1 sequences. The opposite was also observed: one larva recognized as *T. britovi* based on multiplex PCR and 5S rDNA analysis, presented the COX1 sequence identical to *T. spiralis*. The larvae presented combinations of haplotypes, respectively Ts1/TbA, Ts1/TbN and Tb5/TsA.

### 3.2 Geographical distribution

#### 3.2.1 T. spiralis

**Fig. 3:**
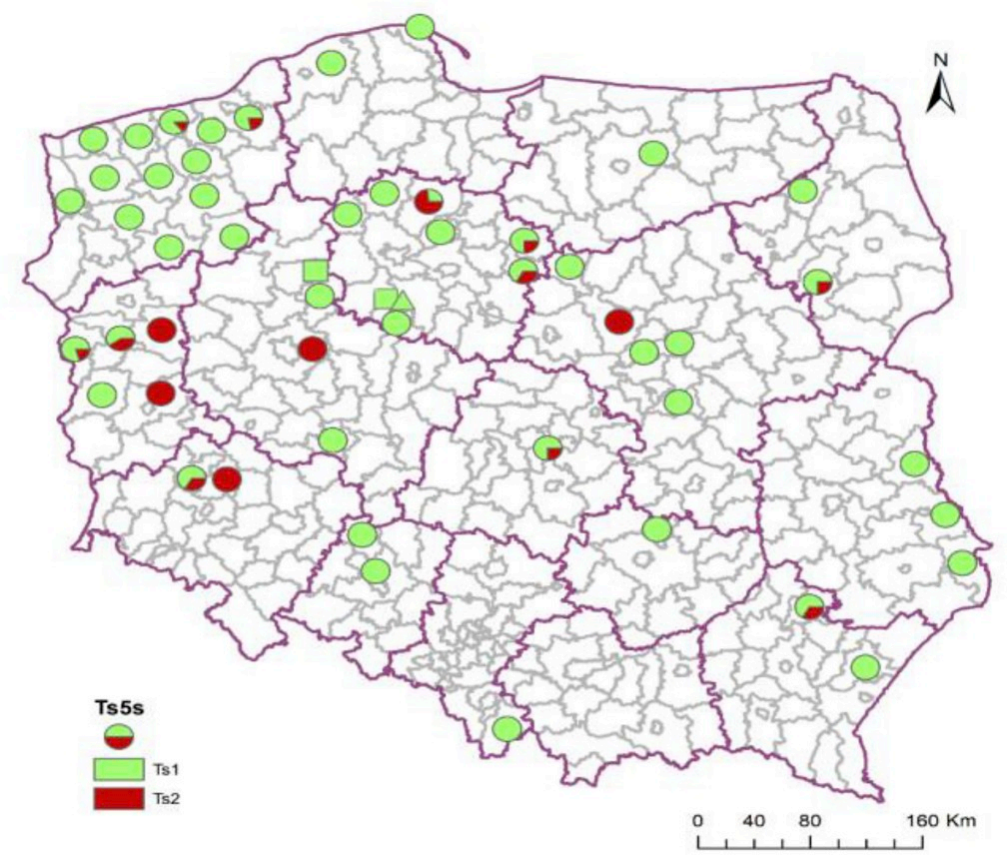
**Geographical distribution of genotypes according to 5S rDNA gene analysis of *T. spiralis* isolates**. Circle – isolates from wild boar, square – isolate from pig, triangle – isolate from rat.

Based on the results of 5S rDNA analysis, the monogenotypic isolates (isolate, in which all tested larvae presented the same genotype) predominated among the isolates studied; 68% belonged to genotype Ts1 and 10% to Ts2. In 11 isolates (22%), both genotypes Ts1 and genotype Ts2 occurred. GenotypeTs1 was discovered in all investigated regions, while the Ts2 genotype was found in central and western Poland (Fig. 3).

**Fig. 4:**
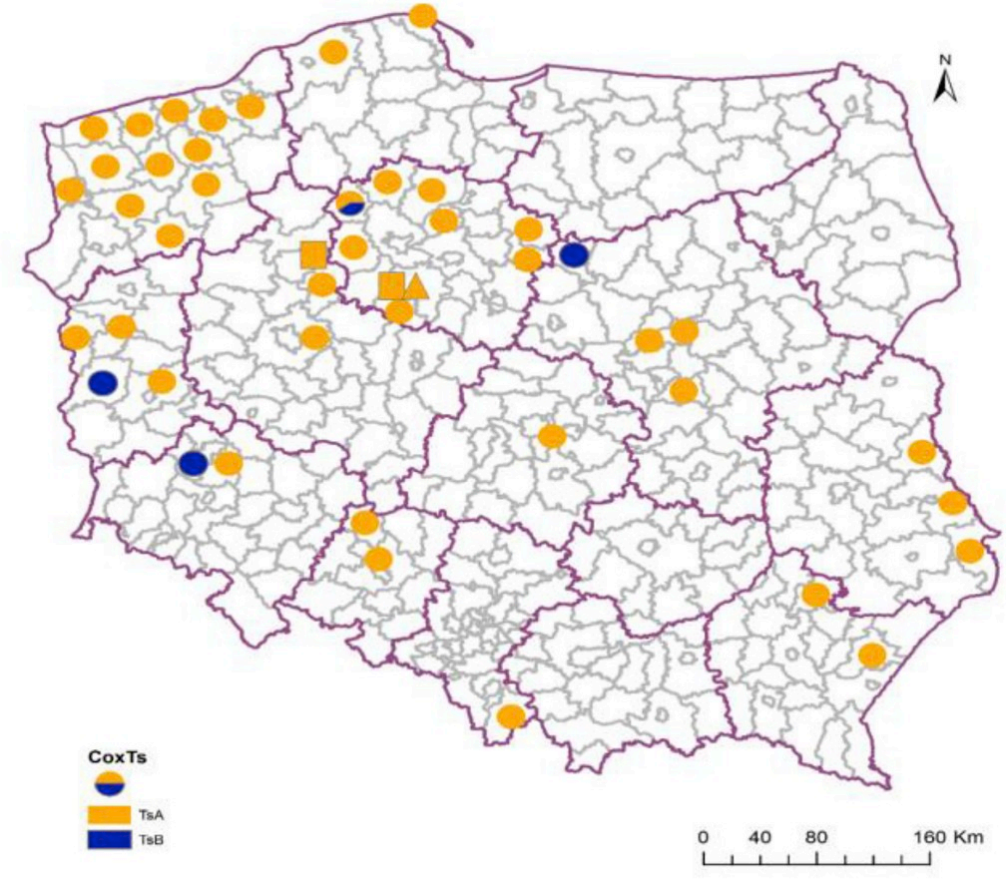
**Geographical distribution of haplotypes according to COX1 gene analysis of *T. spiralis* isolates**. Circle – isolates from wild boar, square – isolate from pig, triangle – isolate from rat.

The analysis of COX1 sequences showed that the investigated isolates of *T. spiralis* were in majority monohaplotypic (isolate in which all tested larvae presented the same haplotype). Only one isolate consisted of larvae of both TsA and TsB haplotypes (Fig. 4).

#### 3.2.2 T. britovi

**Fig. 5:**
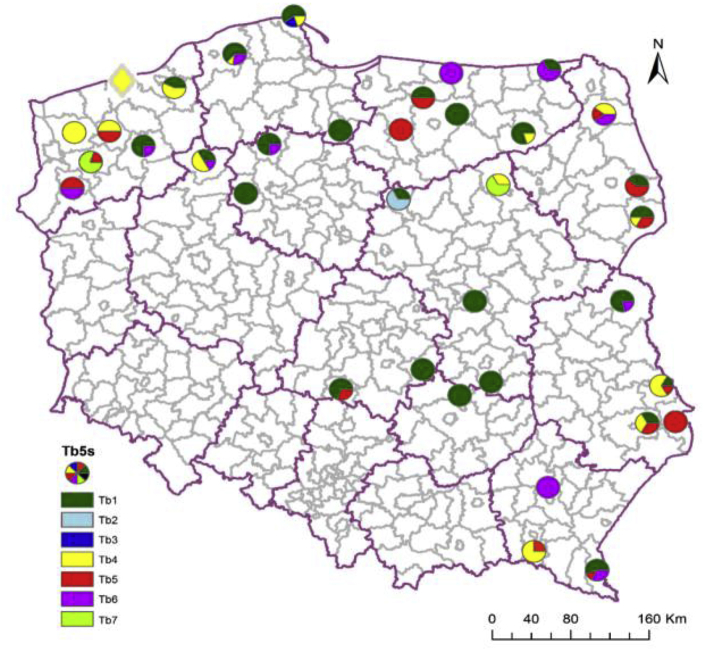
**Geographical distribution of genotypes according to 5S rDNA gene analysis of *T. britovi* isolates.** Circle – isolates from wild boar, rhombus – isolate from red fox. (For interpretation of the references to colour in this figure legend, the reader is referred to the Web version of this article.)

The results of 5S rDNA analysis showed that among isolates of *T. britovi*, the most frequent monogenotypic isolates consisted of larvae representing Tb1 (38%), which were found in six studied regions. Monogenotypic isolates of other genotypes were rare (1 case of Tb4, and 2 cases of Tb2 and Tb6). The remaining isolates consisted of larvae of different genotypes. Among these isolates, larvae carrying Tb4, Tb5 and Tb6 genotypes were most abundant (Fig. 5).

**Fig. 6:**
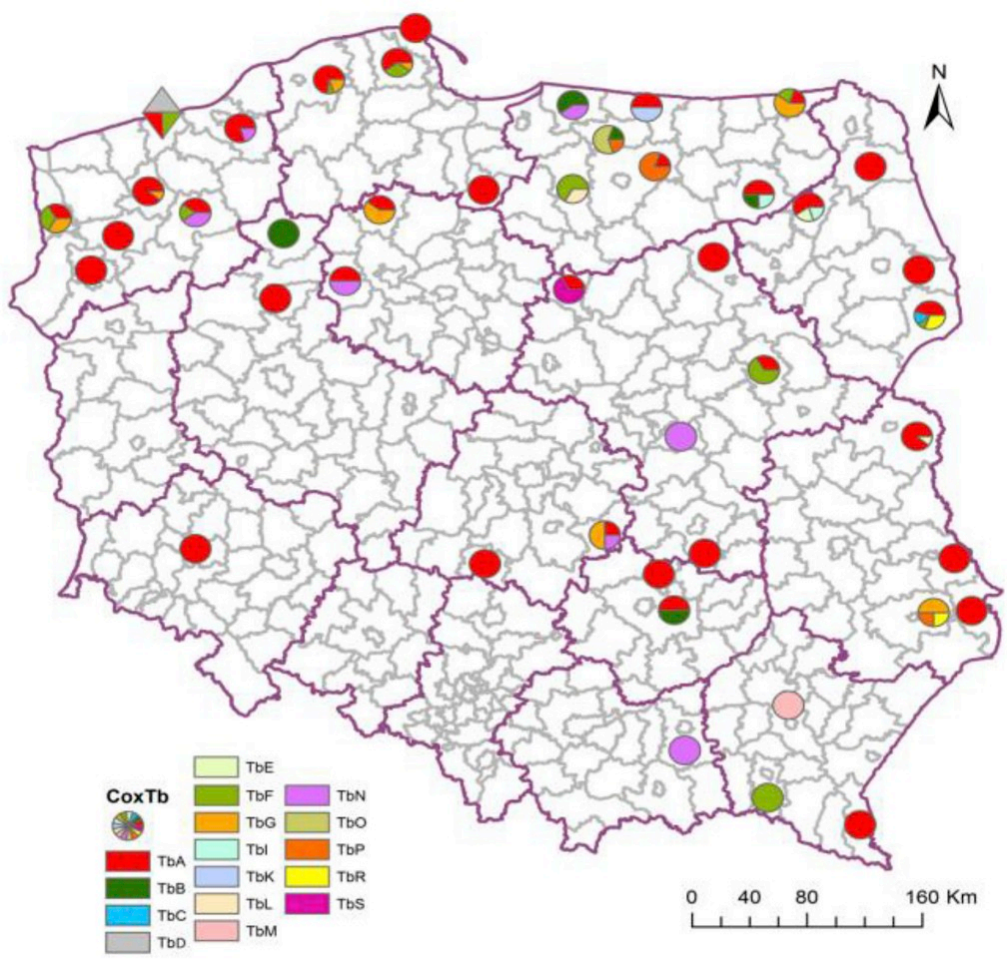
**Geographical distribution of haplotypes according to COX1 gene analysis of *T. britovi* isolates.** Circle – isolates from wild boar, rhombus – isolate from red fox. (For interpretation of the references to colour in this figure legend, the reader is referred to the Web version of this article.)

The obtained results of *T. britovi* analysis showed that 20 isolates consisted of larvae representing one single haplotype (monohaplotypic isolates), in the remaining 24 isolates, larvae of different haplotypes were found. Of all isolates, 34% were isolates composed of TbA haplotype larvae, two isolates consisted of TbN haplotype larvae, one isolate with larvae of TbB and one with larvae of TbM haplotype. Most isolates, however, were composed of larvae of different haplotypes (Fig. 6).

### 3.3 Statistical analysis

#### 3.3.1 Overview

Complete data regarding haplotype, geographical origin, host type and larval counts were available for in total 385 *Trichinella* muscle larvae from 105 isolates; 181 *T. britovi* and 204 *T. spiralis*, originating from 15 regions. In total, 38 *T. britovi* haplotypes have been identified and four for *T. spiralis*, besides three hybrid haplotypes.

Haplotype variation among *T. spiralis* larvae was limited: only two out of 204 (0.98%) *T. spiralis* haplotypes from 12 regions were unique and both were hybrids (Ts1/TbA and Ts1/TbN). Variation among *T. britovi* larvae was considerably higher: 26 *T. britovi* haplotypes (14.4%) were unique among 181 *T. britovi* larvae from 10 regions; another 14 haplotypes (7.7%) shared the same type with an isolate from one other region. Seven haplotypes shared the same type with two isolates from two different regions. Table 7 gives a comprehensive overview of retrieved haplotypes per region for both *Trichinella* species. Each row represents one region and 38 following columns represent haplotypes, which can be read as a unique barcode for each region, representing haplotypes retrieved from 1 to 7 isolates. Macro-regions (according to NUTS 1 division) to which regions belong are shown, as well as the total numbers of haplotypes per macro-region. The obtained barcodes differed between both regions and macro-regions to which they belong.

**Table 7:**
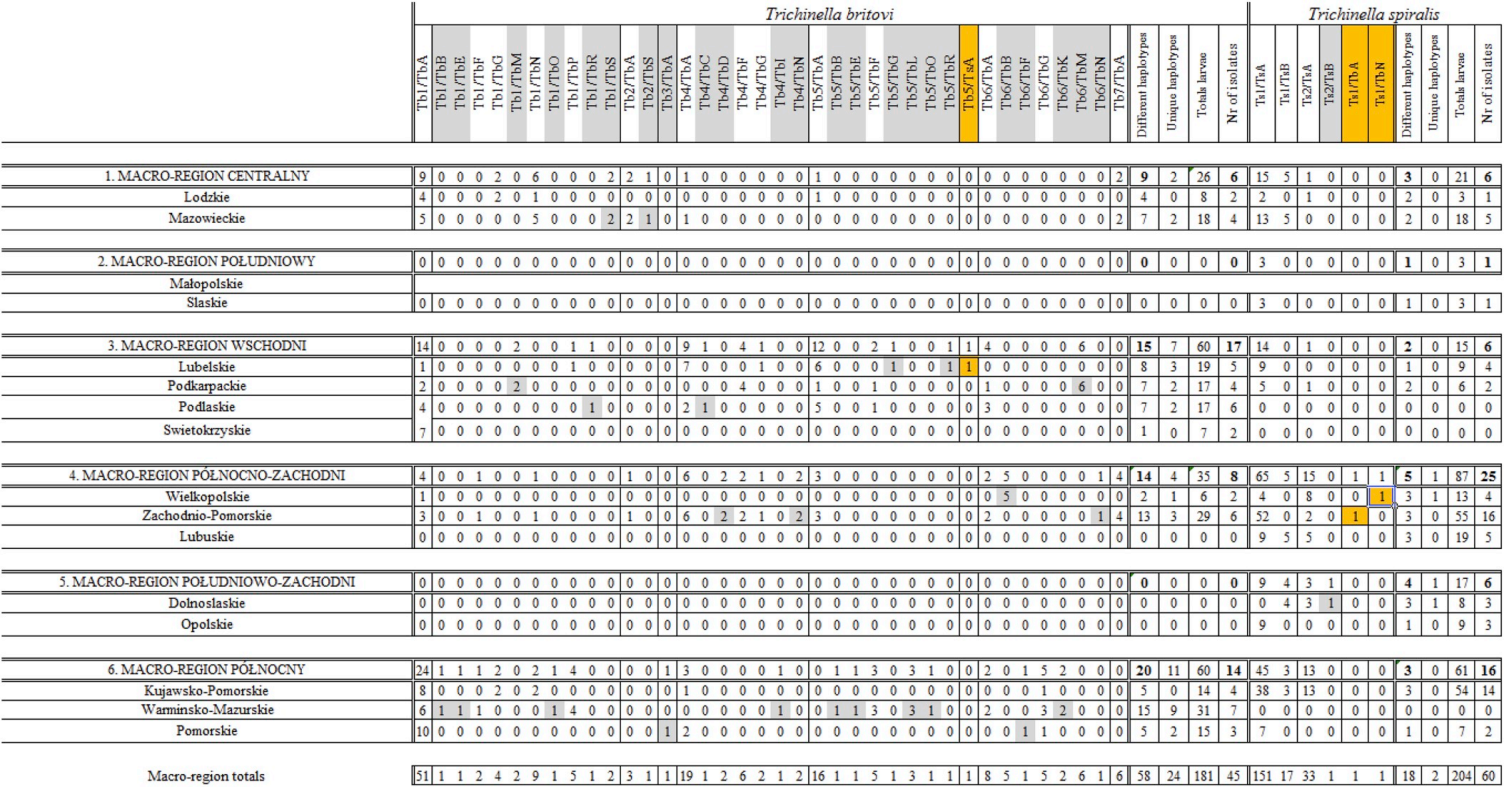
**Overview of *Trichinella* haplotypes per region**. Unique haplotypes are indicated in grey shading. Macro-region names and total numbers per region are indicated horizontally between double lines. Golden shading indicates hybrid *Trichinella* larvae.

#### 3.3.2 Heatmaps

**Fig. 7:**
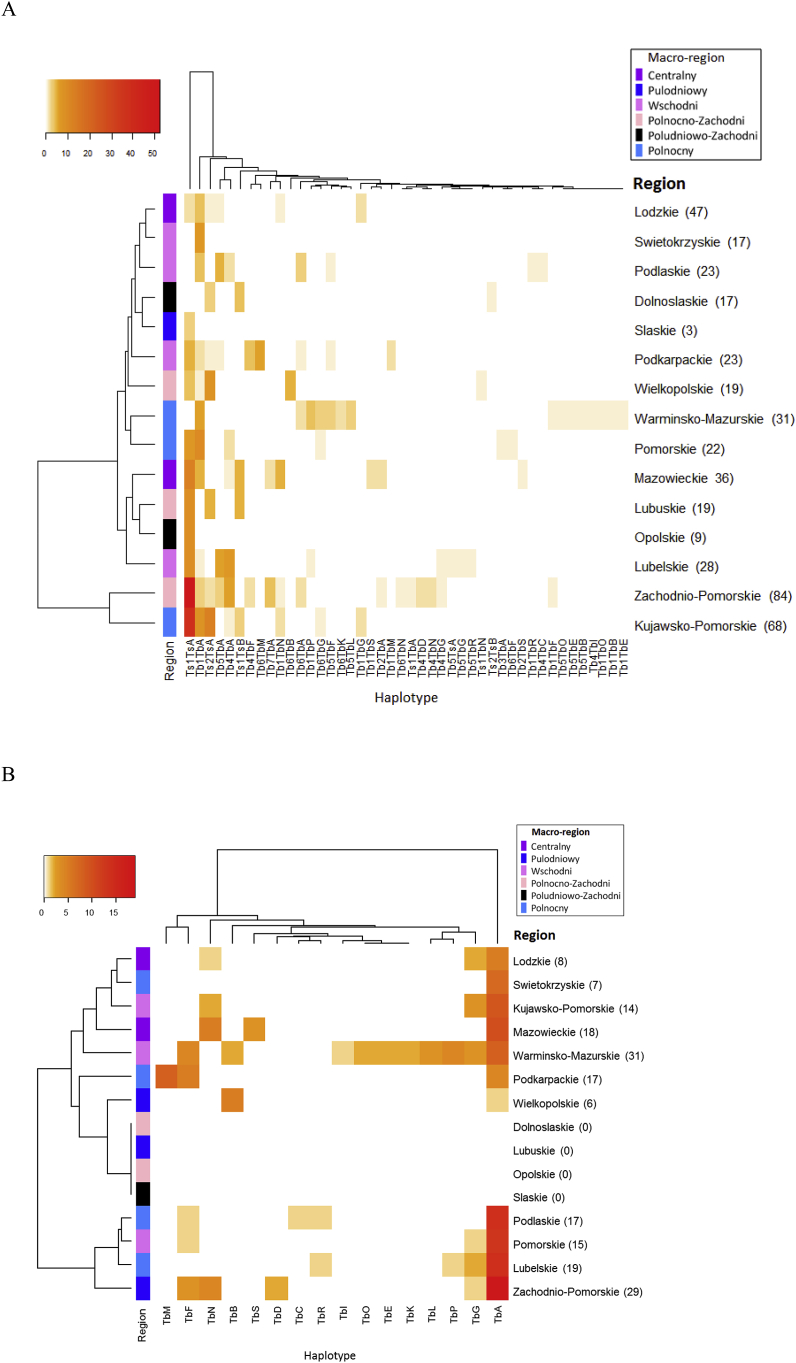
**Heatmap showing *Trichinella* spp. haplotype richness and abundance per region.** Color intensity relates to number of larvae (abundance) per haplotype. The number of tested larvae is shown between brackets behind region names. Dendrograms are derived by Euclidian distance measure, showing correlations between larval richness and abundance (horizontal) and haplotype richness and abundance per region (vertical). The vertical bar left represents macro-regions to which regions belong. **A**: heat map based on all haplotypes found in *T. spiralis* and *T. britovi*. **B**: heat map based on mitochondrial subtype of *T. britovi*. Numbers represent number of examined larvae is shown between brackets. (For interpretation of the references to colour in this figure legend, the reader is referred to the Web version of this article.)

Dendrograms resulting from Euclidian distance measure show correlations between larval richness and abundance (horizontal axis), and haplotype richness and abundance per region (vertical axis) (Fig. 7).

The analysis presented in heatmap A showed that regions Zachodniopomorskie and Kujawsko–Pomorskie clustered into one clade. Mazowieckie, Lubuskie, Opolskie and Lubelskie allocated into a second cluster. Both clusters differed from the other regions, based on larval richness and abundance. Heatmap B shows the results based on *T.britovi* COX1 haplotypes; the dispersion of regions was different compared to heatmap A. Here, Podlaskie, Pomorskie, Lubelskie and Zachodniopomorskie clustered into a clade separate from the other regions. The findings indicate that in Kujawsko-Pomorskie and Zachodniopomorskie mostly *T.spiralis* Ts1TsA was discovered, while when considering only COX1 haplotypes of *T.britovi*, the regions with the highest richness were Podlaskie, Pomorskie, Lubelskie and Zachodniopomorskie. Moreover, the heatmaps showed a pattern for each region, based on which regions may be differentiated with regard to the occurrence of particular haplotypes. The most evident example is Warmińsko-Mazurskie which displayed 15 haplotypes and Zachodniopomorskie that showed 16 haplotypes, but only 3 of those were shared by both regions.

#### 3.3.3 PCA analysis

For regions, the first two principal components account for 81% of the variation, and hence give an accurate summary of the data. One of the attractive properties of PCA is that after the transformation is determined, it is still possible to indicate in the graph 1) the original coordinates (i.e. SNP positions), 2) the locations of the entities by which the points were organized (macro-regions, regions), and 3) any other data which is expressible on the original coordinates (for us: genotypes, expressed as SNP patterns).

The PCA biplot for two main purposes were used, first to determine which SNPs are associated with what region, and second to determine if regions or genotype form natural clusters.

**Fig. 8:**
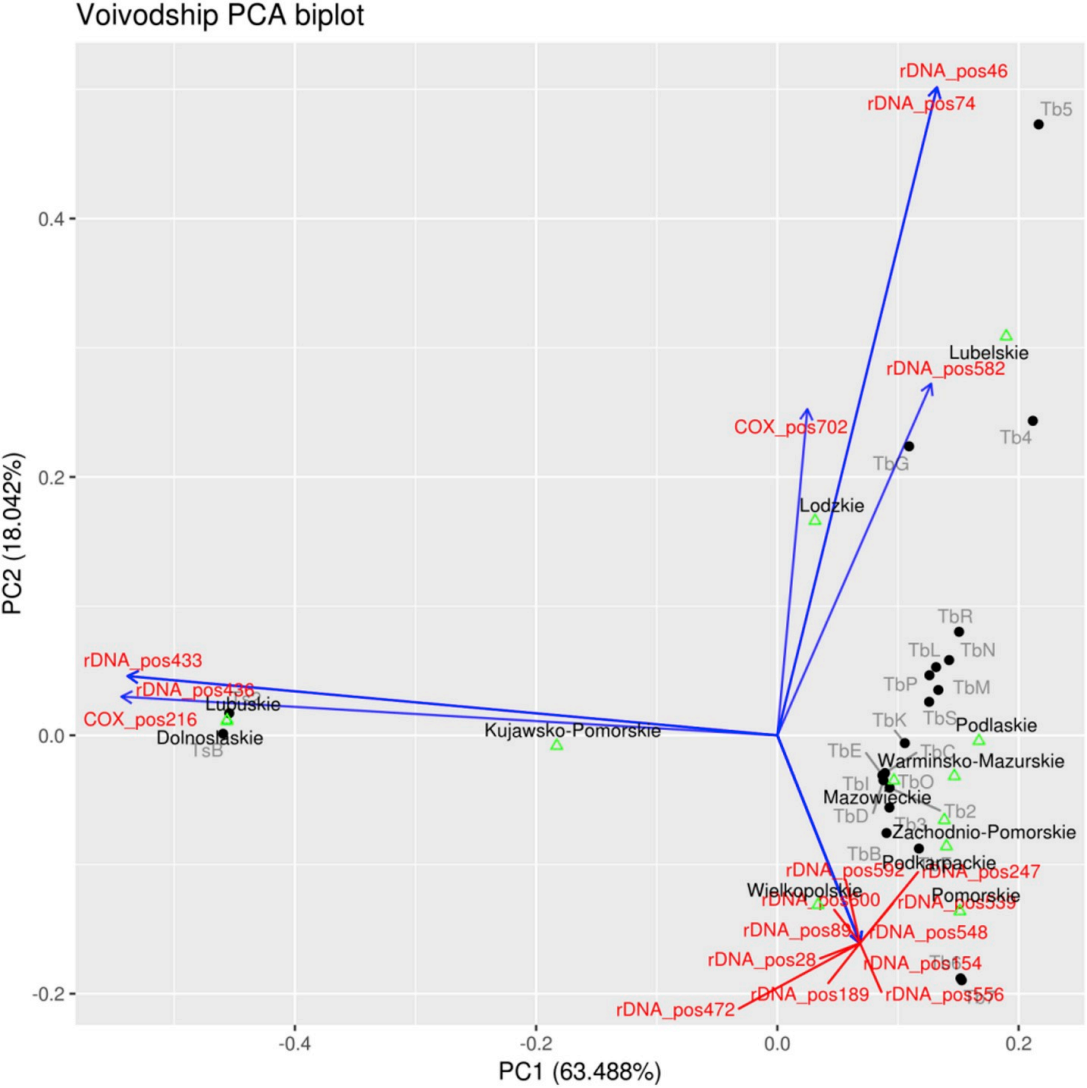
**PCA biplot based on SNPs in both 5S and COX1, according to regions.** The arrow pointing to the bottom right is actually many arrows exactly overlaid (those SNPs convey the same information concerning region variation).

Firstly, we find a clear separation between *T. spiralis* (to the left, TsB and Ts2) and *T. britovi* (to the right, many genotypes) (Fig. 8). Note that TsA, Ts1, TbA and Tb1 lie on the origin, since they have no SNP by definition - they are the baseline types. To the left, we find Dolnośląskie and Lubuskie, that cluster with TsB and Ts2, this is confirmed by checking Table 7, where indeed these species can be seen to be dominant in these regions. Furthermore, also Kujawsko-Pomorskie lies in the direction of TsB and Ts2, but closer to the origin. Indeed, upon closer inspection of Table 7, we do find that Ts1/TsA is much more abundant here than Ts2/TsA. The particular SNPs that lead to this classification are found in COX1 at position 216 and in 5S rDNA at positions 433 and 436. These SNPs should be indicative of Ts2 or TsB, which is easily checked by considering Table 4, Table 5.

Wielkopolskie and Pomorskie regions also lay close together related by the presence of Tb6. Note however that Tb7 which lies close to this cluster, does not appear in it. This may be explained by considering Table 5, where we see that Tb6 and Tb7 share 11 SNPs and differ by one SNP at position 28, even though Tb7 was not actually found in the cluster. This is a possibility, which should be recognized, since the analysis is on the SNP level.

In general, clusters of regions emerge, such as Lubuskie+Dolnośląskie, Łódzkie+Lubelskie, Wielkopolskie+Pomorskie, and a cluster of ‘the rest’. Similarly clusters of geno/haplotypes exist. Those, we can verify independently by checking them against the phylogenetic trees. We may visually assess geno/haplotype clusters. It happens that the COX1 genotypes of *T. britovi* to the right, above the horizontal zero axis, may be identified with the lower cluster in the maximum parsimony tree of Fig. 2. The *T. britovi* species below the horizontal zero axes all belong the upper cluster in Fig. 2. These two clusters can be seen to branch off early in Fig. 2, supported by high bootstrap values, and hence the PCA analysis is in very close concordance with the phylogenetic analysis.

The rightmost part of the figure is not as clear-cut, yet still presents interesting features. Lubelskie and Łódzkie are associated with Tb4, Tb5, and TbG. As outlined above, this is corroborated by Table 7.

## 4 Discussion

The intraspecific genetic variability of *T. spiralis* and *T. britovi* occurring in Poland is substantiated in a limited number of studies (Masny et al., 2012; Franssen et al., 2015). In the present study, the mitochondrial COX1 and the nuclear 5S rDNA gene fragments were used for the molecular characterization of *T. spiralis* and *T. britovi*, in order to determine genetic variability of investigated strains in relation to the geographical origin of their hosts. Based on sequencing, two genotypes (Ts1 and Ts2) were identified in *T. spiralis* 5S rDNA, and two haplotypes in COX1 (TsA and TsB). In contrast, *T. britovi* showed a considerably higher level of variability with 7 genotypes (Tb1–Tb7), based on 17 single nucleotide variations (SNVs) among 5S rDNA sequences, and 16 haplotypes based on 20 SNVs among COX1 sequences (TbA – TbS). Most of SNVs were assessed as SNPs besides two, which were found in less than 1% of investigated *T.spiralis* and *T.britovi* larvae. However, it should be underlined that SNVs, which were not statistically supported to be SNPs, could be assessed as SNPs in the future when appearing in more than 1% of analyzed population. The findings of the present study are similar to those obtained by Franssen et al. (2015) based on isolates from wild boars and red foxes from Poland and Latvia. In that study, identical haplo/genotypes of *T. spiralis* (COX1 and 5S rDNA) were described, and six genotypes based on 5S rDNA and 14 haplotypes in COX1 of *T. britovi*. Each of those variants have been found in the present study, except TbH and TbJ that were discovered only in Latvian isolates (Franssen et al., 2015). Furthermore, in the current study four additional *T. britovi* haplotypes (TbO, TbP, TbR and TbS) were determined in Polish samples. Our results are corroborated by findings of Rosenthal et al. (2008), who demonstrated genetic homogeneity of *T. spiralis* isolates from outside Asia and at the same time higher intraspecific variability within *T. britovi*, using whole mitochondrial genome sequences (Rosenthal et al., 2008). Sequences of *T. spiralis* 5S rDNA from Chinese isolates (ISS79, ISS80, ISS81, ISS82, https://trichinella.iss.it/) displayed more SNPs than were found in the present study, which confirms higher diversification in *T. spiralis* occurring in Asia compared to European isolates of this species, as pointed out by other researchers (Zarlenga et al., 2006; Rosenthal, 2009)(Zarlenga et al., 2009; Yang et al., 2007).

There are a few nucleotide sequences in Genbank which are 100% identical to haplotypes in our study. *T. spiralis* haplotype TsA has been retrieved from a domestic pig from USA (GU386314) and from wild boars in Russia (KU321693). *T. spiralis* variant TsB has been found in Russian isolates from wild boar (MH119334) and wolf (*Canis lupus*) (KU321694) (Odoyevskaya & Spiridonov, 2015). Only one sequence from Genbank (AY009946) showed 100% identity with *T. spiralis* 5S rDNA genotype Ts1 found in our study. The other 5S rDNA sequences deposited in Genbank (mostly originating from Asia) showed less than 100% identity with both Ts1 and Ts2 genotypes.

The haplotypes of *T. britovi* obtained in the present study were mostly unique and newly deposited in database. Among all COX1 haplotypes obtained in this study, only two displayed 100% coverage and identity with Genbank sequences. *T. britovi* TbA was found to be 100% identical with sequences originating from red foxes from Italy (KM357413.1). *T. britovi* TbS showed 100% identity to nucleotide sequences of *T. britovi* collected from Iranian wild boars (KY464996, KY464997). These examples indicate that various genotypes of *T. britovi* may be more widespread in different regions of world. Moreover, the occurrence of dozens of various genotypes in Poland alone, implies that the genetic variability of the *T. britovi* population may be even higher, taking into account that only two gene fragments have been investigated. It should be noted that the majority of isolates used in the present study (n = 49) was collected from wild boars. Additionally, the numerous SNPs that occurred in both analyzed genes from just one host species (wild boar) may indicate that *T. britovi* probably is richer in genetic variants than expected. Evidence for different genotypes occurring in *T. britovi* isolated from wild boars has also been provided for different genes in other studies e.g. in Spain (Perteguer et al., 2009; Fonseca-Salamanca et al., 2009), Corsica and Sardinia (La Rosa et al., 2018).

The 5S rDNA gene is well suited for molecular identification of *Trichinella* species (van der Giessen et al., 2005). The results of our own studies based on phylogenetic analysis of the obtained sequences confirm this in the case of *T. spiralis*. However, for *T. britovi*, the genotypic variation blurs species identification: genotypes Tb1, Tb2, Tb3, Tb4 and Tb5 clustered within a *T. britovi* clade, together with the *T. britovi* reference sequence from Genbank, while the genotypes Tb6 and Tb7 appeared more closely related to *Trichinella* T9 from Japan. Based on COX1 sequence analysis, *Trichinella* T9 clusters with nine *T. britovi* haplotypes (this present study). It is possible that these haplotypes have common ancestry with T9 or may result from the migration of animals from Asia to the west, and consequent evolution to different haplotypes of parasites.

Combined analysis of both genes (5S rDNA and COX1), showed the occurrence of interspecific hybrids of *T. spiralis*/ *T. britovi*. These hybrids were isolated from three isolates of larvae from wild boars of three different geographical areas. Interspecies cross hybridization between *T. spiralis* and *T. britovi* under natural conditions has been identified earlier in Polish isolates (Franssen et al., 2015), which could be achieved under experimental conditions several years ago by prof. E. Pozio (Franssen et al., 2015) and more recently by Wu et al. (2000). Although hybridization between *T. spiralis* and *T. britovi* is thought to have taken place fairly recently (≤1000 years before present (Rosenthal et al., 2008; La Rosa et al., 2012)), it is difficult to determine what its consequences may be. Hybridization in itself is not unique; *Trichinella nativa*/ *Trichinella* T6 hybrids have been discovered under natural conditions (Dunams-Morel et al., 2012; La Rosa et al., 2003). Phenotypic properties of both species are different and hybridization between *Trichinella* genotypes may result in various changes in genotypic characteristics, but also in their phenotypic traits. Several effects of such introgression events have been proposed, from limitation of gene flow between two lineages, through fixation of hybrids as a distinct biological species, up to the extinction of a less successful lineage in coinfections (Hecht et al., 2016). An example of interspecies hybridization leading to phenotypic advantageous treats is seen in digenean schistosomes infecting humans (*Schistosoma haematobium*), bovines (*Schistosoma bovis*), and sheep, goats and bovines (*Schistosoma curassoni*), where hybridization lead to host range expansion and decreased susceptibility to drug treatment (Huyse et al., 2013; Webster et al., 2013; Sene-Wade et al., 2018).

Finally, the occurrence of mutations in mitochondrial genes and subsequent translations into different amino acids can cause change of protein function, which may result in unpredictable changes of characteristics of such organism. An example is *Caenorhabditis elegans*, of which wild isolates of varying geographic origins may have adapted to environmental challenges over time through mtDNA variation, to modulate critical aspects of their mitochondrial energy metabolism (Dingley et al., 2014).

Higher genetic variability of *T. britovi* compared to *T. spiralis* results probably from the longer evolutionary history of this species in its geographical distribution area, while *T. spiralis* was introduced into Europe far more recently on a geological time scale (Rosenthal et al., 2008, Webb and Rosenthal, 2010). Additionally, *T. britovi* infects a wider range of hosts in sylvatic environments; circulation of these parasites between different hosts may also predispose for mutations in the parasite's genes (La Rosa et al., 2012). The finding of genetic variability in *Trichinella* spp. could be a basis for developing a useful scheme to differentiate isolates from different host/regions. The dominating genotype in the Polish *T. spiralis* population was Ts1 of 5S rDNA and TsA of COX1 in the present study. Out of 4 haplotype combinations of *T. spiralis*, the most abundant was Ts1TsA, which was the most frequent in Zachodniopomorskie and Kujawsko-pomorskie. Although our results do not allow source attribution or identification of geographical origin of *Trichinella* isolates, the collected data showed different haplotype compositions in each region. The most interesting finding was high haplotype richness in a few regions, e.g. Warmińsko-Mazurskie and Zachodniopomorskie regions, where 15 and 16 haplotypes were discovered, respectively. The high variability in *Trichinella* spp. found in this region could result from several factors. The most striking geographic characterization of these regions are the high number of lakes and wetlands. Such conditions predispose to attract a wide range of fauna, and among it animal species which can be infected by *Trichinella* spp. This is confirmed by the high number and density of population of wild boars and red foxes compared to other regions in Poland (https://www.pzlow.pl/, 2018.02.06). The occurrence of various host types in a given area causes mixtures of parasite populations which may result in genetic variability. Moreover, the Warmińsko-Mazurskie region, which is closest to the Baltic States, displayed the highest richness in *T. britovi* haplotypes. Previously, a high variation among Latvian *T britovi* isolates was discovered (Franssen et al., 2015). Lithuania, Latvia and Estonia report predominantly *T. britovi* infection in wildlife (Malakauskas et al., 2007) and migration of animals like red foxes and raccoon dogs carrying their *T.britovi* variants into Poland may contribute to haplotypes richness.

The higher *T.britovi* genetic variability offers an opportunity to differentiate isolates based on haplotype composition and geographical origin of their hosts. The heatmaps presented in this paper could be a helpful tool to achieve this aim in the future, however more work has to be done to enrich the haplotypes database for the Polish regions. Heatmaps are commonly used in statistical analysis of bacterial studies, for example to compare abundance and richness of particular pathogens in different types of samples (Young et al., 2015). Furthermore, the Principal Component Analysis presented in the current study may help with future epidemiological investigations to recognize isolate origin, based on SNPs discovered in the larvae. The PCA biplot in the current study showed that some neighboring provinces clustered, which may confirm that in some cases, geographical origin of the host correlates with particular haplotype occurring in a given isolate.

In general, the variants determined in the current study were highly dispersed in Poland; investigating higher numbers of isolates might help to correlate variants with their geographical distribution. If such relationships could be substantiated, this would allow source attribution of *Trichinella* outbreak cases. Additionally, different genetic markers could be used, e.g. microsatellites analysis, which provided promising results in relation to their geographical origin in another study (La Rosa et al., 2018).

## 5 Conclusion

*T. spiralis* and *T. britovi* present different levels of genetic variability in their 5S rDNA and COX1 partial gene sequences. Circulating in more than 75% of infected wild boars, *T. spiralis* was found to be very homogenous, exhibiting only two haplotypes in both genes. *T. britovi*, circulating at lower prevalence in wild boar (<20%), displayed considerably higher genetic variation in both genes. These results may be useful to differentiate isolates of *T. britovi* according to geographical origin, aiding in source attribution at outbreaks where *T. britovi* is involved. However, more research is needed to substantiate this method in the future.

## Funding

The results described in the article are a part of the PhD thesis entitled “Analysis of the genetic structure of *Trichinell*a nematodes occurring in Poland and its application in epidemiological investigations” by Ewa Bilska-Zając supported by the Polish Ministry of Science and Higher Education (grant S260 - An attempt to determine the essential vectors of *Trichinella* in sylvatic and synanthropic environments).

## Conflicts of interest

The authors declare that there is no conflict of interest.
